# Prevalence and Correlates of Cryptococcal Antigen Positivity Among AIDS Patients — United States, 1986–2012

**Published:** 2014-07-11

**Authors:** Jennie McKenney, Rachel M. Smith, Tom M. Chiller, Roger Detels, Audrey French, Joseph Margolick, Jeffrey D. Klausner

**Affiliations:** 1 Department of Epidemiology, Fielding School of Public Health, University of California–Los Angeles; 2 Division of Foodborne, Waterborne, and Environmental Diseases, National Center for Emerging and Zoonotic Infectious Diseases, CDC; 3 Division of Infectious Diseases, John H. Stroger, Jr. Hospital of Cook County, Chicago, IL; 4 Department of Molecular Microbiology and Immunology, Johns Hopkins University, Baltimore, MD; 5 Department of Medicine, Division of Infectious Diseases, David Geffen School of Medicine, University of California–Los Angeles

Cryptococcal meningitis (CM) is one of the leading opportunistic infections associated with human immunodeficiency virus (HIV) infection (1). The worldwide burden of CM among persons living with HIV/acquired immunodeficiency syndrome (AIDS) was estimated in 2009 to be 957,900 cases, with approximately 624,700 deaths annually (1). The high burden of CM globally comes despite the fact that cryptococcal antigen (CrAg) is detectable weeks before the onset of symptoms, allowing screening for cryptococcal infection and early treatment to prevent CM and CM-related mortality (2). However, few studies have been conducted in the United States to assess the prevalence of cryptococcal infection. To quantify the prevalence of undiagnosed cryptococcal infection in HIV-infected persons in the United States during 1986–2012, stored sera from 1,872 participants in the Multicenter AIDS Cohort Study and the Women’s Interagency HIV Study with CD4 T-cell counts <100 cells/*μ*L were screened for CrAg, using the CrAg Lateral Flow Assay (LFA) (Immy, Inc.). This report describes the results of that analysis, which indicated the overall prevalence of CrAg positivity in this population to be 2.9% (95% confidence interval [CI] = 2.2%–3.7%).

CrAg is detectable in serum a median of 3 weeks before the onset of symptoms of CM, making screening for serum CrAg and subsequent treatment of those with a positive test result a potential means of reducing CM-related mortality (2). In 2011, the World Health Organization declared early antiretroviral therapy (ART) initiation the most important and cost-effective preventive strategy to reduce the incidence and high mortality associated with CM. They further declared (as a conditional recommendation, given the low quality of evidence) that routine screening of and treatment for cryptococcal antigenemia might be considered before ART initiation for ART-naïve adults with a CD4 T-cell count <100 cells/*μ*L in areas with a high prevalence (approximately 3%) of cryptococcal antigenemia.*

There are few data from the United States on the prevalence of cryptococcal antigenemia. To estimate the prevalence, stored sera collected from 1986–2012 from the Multicenter AIDS Cohort Study and the Women’s Interagency HIV Study were screened for CrAg. To be eligible for the study, specimens needed to come from study participants who were HIV-infected, had a CD4 T-cell count <100 cells/*μ*L, and had ≥0.5mL of stored serum available for testing. Sera from participants on and off ART were eligible for screening. Serum specimens randomly selected from those eligible were tested using the CrAg LFA. This is a Food and Drug Administration–cleared CrAg detection test, which demonstrated a sensitivity of 100% for the detection of CrAg in archived sera from 704 HIV-infected patients hospitalized for acute respiratory illness in Thailand (3). Chi-square and Student’s t-tests were performed to test differences between those with positive and negative CrAg test results.

A total of 1,872 serum specimens from the Multicenter AIDS Cohort Study and the Women’s Interagency HIV Study were screened. The median age of the study population was 39 years (range = 20–70 years). Of the 1,872 specimens, 55 (2.9% [CI = 2.2%–3.7%]) were positive for CrAg. No significant differences were observed in the proportion of CrAg-positive specimens by specimen collection year, age, sex, study location, level of education, or race/ethnicity, except that persons of “other” ethnicity (i.e., not white, black, or Hispanic) had a prevalence of 6.4% (CI = 3.9%–10.3%) (Table). Persons with a CD4 count >50 cells/*μ*L were less likely to be CrAg-positive compared with persons with a CD4 count ≤50 cells/*μ*L (1.7% [CI = 1.1%–2.7%] and 4.3% [CI = 3.2%–5.9%], respectively).

## Discussion

Results from this U.S. study indicated a 2.9% (CI = 2.2%–3.7%) prevalence of cryptococcal antigenemia in advanced AIDS patients. However, within certain subgroups, CrAg prevalences were higher. Existing prevalence data for CrAg antigenemia are mostly from resource-limited settings and range from as low as 2% in northern Vietnam (4) to 21% in Benin City, Nigeria (5). South Africa, which began piloting CrAg screening in HIV-infected patients with CD4 T-cell counts <100 cells/*μ*L, and treatment of those with positive CrAg tests in 2012, reported a prevalence of 5% in the 12 months after the initiation of that program.†

In the United States, early access to ART has reduced the morbidity and mortality attributable to CM (6). However, the incidence of CM among those with AIDS remains between two and seven cases per 1,000 persons, with a mortality rate as high as 12% (7). Late presentation to care among HIV-infected persons remains a problem in the United States, with 38% of persons newly diagnosed with HIV infection receiving an AIDS diagnosis concurrently or within the next year; these “late presenters” are more likely to be diagnosed and to die from preventable opportunistic infections, including CM.§

The devastating health effects of CM are compounded by the substantial clinical and financial burdens incurred when treating the disease. Treatment of CM requires toxic drugs, such as amphotericin B, repeated procedures, such as lumbar punctures, and close monitoring, all of which require hospitalization. In the United States, the mean hospital stay for persons diagnosed with CM is 14.7 days, costing, on average, $50,000 (U.S.) for the entire hospital stay, and totaling $301.6 million spent per year treating CM (7). Those costs do not include the costs associated with complications, relapse, or outpatient care once a patient has been discharged. A proportion of CM cases are preventable, as was shown in an observational study conducted in Uganda: among ART-naïve, HIV-infected patients, with a CD4 T-cell count <100 cells/*μ*L, the number needed to screen to prevent one case of CM was 11.3, and the number needed to screen to prevent one death was 15.9 (8). Therefore, screening would have tangible cost benefits because fewer cases of CM would mean less money spent on treating the disease. Several studies have examined the cost-effectiveness of CrAg screening. In a model using South African data, two screen-and-treat strategies, CrAg screening followed by high dose of fluconazole and CrAg screening followed by lumbar puncture, were compared to the standard of care, no screening (9). Both screen-and-treat strategies were more cost-effective than the standard of care. The least costly strategy was screening followed by high-dose fluconazole treatment, which was more cost-effective than the standard of care at prevalence levels ≥0.6%, lower than the estimated prevalences presented in this report (9).

The implementation of CrAg screening programs has begun in several countries, including South Africa, Rwanda, and Mozambique. However, in most of the world, including the United States, no recommendation for CrAg screening exists, despite the fact that a large number of HIV-infected persons present late to care every year, many of whom might benefit from CrAg screening. In 2013, the HIV Medicine Association of the Infectious Disease Society of America did not recommend routine screening for CrAg, but stated that screening “may be considered in selected patients with CD4 cell counts <50 cells/*μ*L” (10).

Inclusion of CrAg screening in a “late presenter” care package for HIV-infected persons in the United States has several advantages. Treatment of patients with isolated cryptococcal antigenemia has been shown to prevent the development of CM and CM-related death (7). Additionally, because those with cryptococcal antigenemia would likely undergo a lumbar puncture to be evaluated for CM, screening would likely improve the early diagnosis of CM. Early diagnosis of CM would translate into a reduction in CM-related deaths because patients who receive early antifungal treatment have been shown to have better outcomes than those who receive delayed treatment (8).

What is already known on this topic?

Cryptococcal meningitis is the leading opportunistic infection worldwide among persons infected with human immunodeficiency virus (HIV). No recent study of the prevalence of cryptococcal antigenemia among HIV-infected persons has been conducted in the United States.

What is added by this report?

Serum specimens from HIV-infected persons with low CD4 T-cell counts enrolled in studies in the United States during 1986–2012 were screened for cryptococcal antigen using a new lateral flow assay. The prevalence of cryptococcal antigenemia was 2.9% (95% confidence interval = 2.2%–3.7%).

What are the implications for public health practice?

The prevalence of cryptococcal infection among patients with advanced acquired immunodeficiency syndrome (AIDS) in the United States is high enough that screening for cryptococcal antigenemia might save lives and be cost-effective.

This study has several limitations. First, the study spanned more than two decades and includes many participants who were diagnosed before the advent of highly active ART. Predictors of cryptococcal infection might be different among groups before and after the introduction of highly active ART. However, when CrAg results were compared by various periods and current ART use, no differences were found. Second, no lumbar puncture results were available to help determine what proportion of patients with a positive serum CrAg result had prior or incident CM. Finally, this study was conducted using participants from the Multicenter AIDS Cohort Study and Women’s Interagency HIV Study, both of which include participants that might not be representative of the U.S. HIV/AIDS population.

These findings provide national CrAg seroprevalence data for HIV-infected persons with CD4 T-cell count <100 cells/*μ*L in the United States. The findings show the prevalence to be above the published cost-effectiveness thresholds in resource-limited countries and on par with many other countries that are currently pursuing programs of routine CrAg screening and treatment. Based on these data, updated strategies for the prevention of CM among AIDS patients in the United States should be considered.

## Figures and Tables

**TABLE:** Selected characteristics of participants (N = 1,872) with tested specimens and cryptococcal antigen prevalence — Multicenter AIDS Cohort Study and Women’s Interagency HIV Study, United States, 1986–2012

			Cryptococcal antigen prevalence	
			
Characteristic	No.	(%)	(%)	(95% CI)
**Total**	**1,872**	**(100)**	**2.9**	**(2.2–3.7)**
**Sex**				
Male	989	(53.3)	2.6	(1.7–3.8)
Female	866	(46.7)	3.3	(2.3–4.7)
**Study location**				
Baltimore, MD	241	(12.9)	3.0	(1.5–6.0)
Bronx, NY	183	(9.9)	4.3	(2.2–8.4)
Brooklyn, NY	162	(8.7)	1.9	(0.6–5.3)
Chicago, IL	342	(18.4)	0.5	(0.1–2.8)
District of Columbia	123	(6.6)	4.0	(1.7–9.1)
Los Angeles, CA	514	(27.7)	3.3	(2.1–5.2)
Pittsburgh, PA	187	(10.1)	4.2	(2.1–8.3)
San Francisco, CA	103	(5.6)	3.9	(1.5–9.5)
**Race/Ethnicity**				
White	849	(45.9)	2.5	(1.6–3.8)
Black	651	(35.2)	2.5	(1.5–4.0)
Hispanic	117	(6.3)	1.7	(0.5–6.7)
Other	234	(12.6)	6.4	(3.9–10.3)
**Education**				
High school or less	790	(42.9)	3.8	(2.7–5.4)
College	773	(42.1)	2.5	(1.6–3.8)
Graduate school	275	(14.9)	1.5	(0.6–3.9)
**Period of specimen collection**				
1986–1990	485	(26.3)	2.1	(1.1–3.8)
1991–1995	620	(33.7)	3.6	(2.4–5.3)
1996–2000	255	(13.9)	1.6	(0.6–4.0)
2001–2005	288	(15.6)	3.5	(1.9–6.3)
2006–2012	193	(10.5)	3.6	(1.8–7.3)
**Age group (yrs)**				
20–30	188	(10.0)	2.1	(0.8–5.4)
31–40	775	(41.4)	3.1	(2.1–4.6)
41–50	556	(29.7)	2.5	(1.5–4.2)
51–60	142	(7.6)	4.2	(2.0–8.9)
≥61	18	(9.6)	5.6	(1.0–25.8)
**CD4 count**				
>50 cells/*μ*L	992	(53.0)	1.7	(1.1–2.7)
≤50 cells/*μ*L	881	(47.1)	4.3	(3.2–5.9)
**Receiving ART at time of specimen collection**				
Yes	1,047	(55.9)	1.4	(1.0–2.1)
No	743	(39.7)	1.4	(1.0–2.1)

**Abbreviations:** CI = confidence interval; ART = antiretroviral therapy.

## Footnotes

* Additional information available at http://www.who.int/hiv/pub/cryptococcal_disease2011.

† Additional information available at http://www.nicd.ac.za/assets/files/Monthly%20NICD%20Surveillance%20Report%20-%20January%202013.pdf.

§ Additional information available at http://www.cdc.gov/mmwr/preview/mmwrhtml/mm5824a2.htm.
